# Pathophysiology of Ca_v_1.3 L-type calcium channels in the heart

**DOI:** 10.3389/fphys.2023.1144069

**Published:** 2023-03-21

**Authors:** Sahil Zaveri, Ujala Srivastava, Yongxia Sarah Qu, Mohamed Chahine, Mohamed Boutjdir

**Affiliations:** ^1^ Cardiovascular Research Program, VA New York Harbor Healthcare System, New York, NY, United States; ^2^ Department of Medicine, Cell Biology and Pharmacology, State University of New York Downstate Health Sciences University, Brooklyn, New York, NY, United States; ^3^ Department of Cardiology, New York Presbyterian Brooklyn Methodist Hospital, New York, NY, United States; ^4^ CERVO Brain Research Center, Institut Universitaire en Santé Mentale de Québec, Québec, QC, Canada; ^5^ Department of Medicine, Faculté de Médecine, Université Laval, Quebec, QC, Canada; ^6^ Division of Cardiology, Department of Medicine, NYU Grossman School of Medicine, New York, NY, United States

**Keywords:** calcium channel, sinoatrial node dysfunction, atrial fibrillation, heart failure, protein kinase regulation

## Abstract

Ca^2+^ plays a crucial role in excitation-contraction coupling in cardiac myocytes. Dysfunctional Ca^2+^ regulation alters the force of contraction and causes cardiac arrhythmias. Ca^2+^ entry into cardiomyocytes is mediated mainly through L-type Ca^2+^ channels, leading to the subsequent Ca^2+^ release from the sarcoplasmic reticulum. L-type Ca^2+^ channels are composed of the conventional Ca_v_1.2, ubiquitously expressed in all heart chambers, and the developmentally regulated Ca_v_1.3, exclusively expressed in the atria, sinoatrial node, and atrioventricular node in the adult heart. As such, Ca_v_1.3 is implicated in the pathogenesis of sinoatrial and atrioventricular node dysfunction as well as atrial fibrillation. More recently, Ca_v_1.3 *de novo* expression was suggested in heart failure. Here, we review the functional role, expression levels, and regulation of Ca_v_1.3 in the heart, including in the context of cardiac diseases. We believe that the elucidation of the functional and molecular pathways regulating Ca_v_1.3 in the heart will assist in developing novel targeted therapeutic interventions for the aforementioned arrhythmias.

## Introduction

Cardiac excitation-contraction coupling is a process where electrical excitation of the cardiomyocyte leads to a muscular contraction in the heart (Bers, 2002). L-type Ca^2+^ channels play an essential role in excitation-contraction coupling by mediating Ca^2+^ influx and membrane excitability (Bodi et al., 2005; Striessnig et al., 2014; Catterall et al., 2020). These Ca^2+^ channels are modulated by small concentrations of different chemical classes of Ca^2+^ antagonists, including dihydropyridines (Berjukow et al., 2000; Tang et al., 2016). There are four types of L-type Ca^2+^ channels: Ca_v_1.1, Ca_v_1.2, Ca_v_1.3, and Ca_v_1.4 (Berjukow et al., 2000; Berger and Bartsch, 2014; Striessnig et al., 2014). The Ca_v_1.1 and Ca_v_1.4 channels are restricted to the skeletal muscle and retina/immune cells, respectively. However, Ca_v_1.2 is more widely expressed in the heart/smooth muscle, neurons (somatodendritic), and endocrine cells, while Ca_v_1.3 is expressed in the heart, neurons (somatodendritic), endocrine cells, and sensory cells (Striessnig et al., 2014; Mesirca et al., 2015).

**FIGURE 1:**
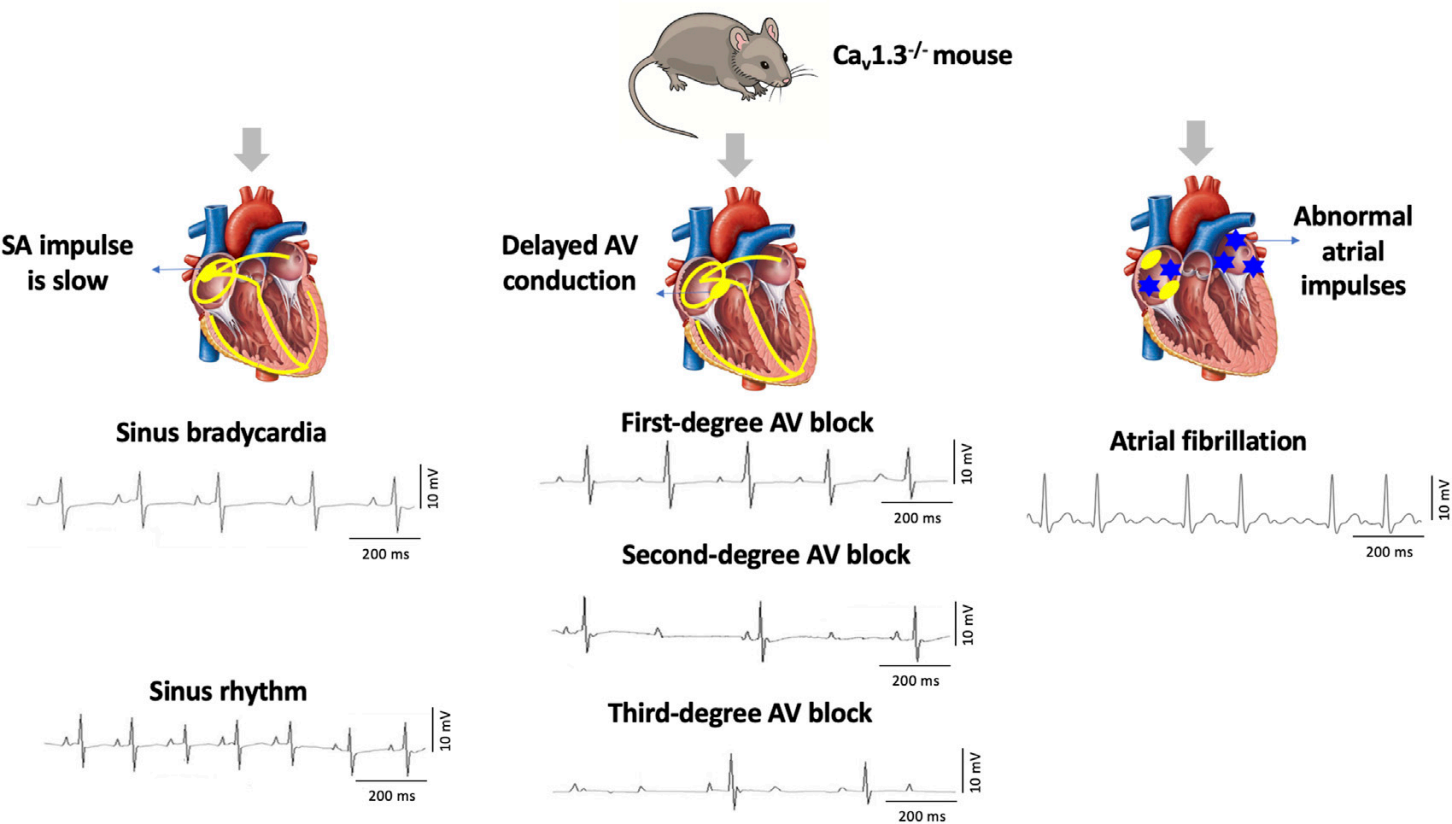
Deletion of Ca_v_1.3 in mice leads to electrographic abnormalities. The absence of Ca_v_1.3 in mice leads to the development of sinoatrial (SA) and atrioventricular (AV) node dysfunction leading to sinus bradycardia and first-degree, second-degree, and third-degree AV block as well as atrial fibrillation (AF).

Ca_v_1.3, the focus of this review, was initially thought to be of neuroendocrine origin (Qu et al., 2005b; Zhang et al., 2005; Lu et al., 2015). However, it was subsequently discovered in the adult heart with distinct expression exclusively in the supraventricular tissues (atria, sinoatrial node, atrioventricular node) and not in the ventricles (Mangoni et al., 2003; Qu et al., 2005a). Genetic deletion of Ca_v_1.3 in mice (Ca_v_1.3^−/−^) causes congenital deafness, sinus bradycardia, and various degrees of atrioventricular (AV) block consistent with region-specific expression (Mangoni et al., 2003; Hu et al., 2004; Qu et al., 2005a). Furthermore, Ca_v_1.3^−/−^ mice display impaired Ca^2+^ homeostasis associated with atrial fibrillation (AF) (Figure 1) (Mancarella et al., 2008). Interestingly, loss of Ca_v_1.3 function in humans was associated with sinoatrial node dysfunction and deafness (SANDD) syndrome with a cardiac and auditory phenotype similar to Ca_v_1.3^−/−^ mice (Baig et al., 2011; Liaqat et al., 2019; Torrente et al., 2020).

Numerous neurotransmitters regulate Ca_v_1.3 in the heart. Phosphorylation of the channel by cAMP-dependent protein kinase A (PKA) is at serine residues located at positions 1743 and 1816 of the C-terminus (Mitterdorfer et al., 1996). Protein kinase C (PKC) also plays a vital role in regulating Ca_v_1.3 in an isozyme-specific manner, with the regulation site being a serine residue located at position 81 of the N-terminal domain (Baroudi et al., 2006). When calmodulin-dependent protein kinase II (CaMKII) is co-expressed with densin, which binds to Ca_v_1.3, it facilitates the increase of Ca^2+^ current (I_CaL_) as a result of high-frequency stimulation (Maier and Bers, 2002; Jenkins et al., 2010; Tokumitsu and Sakagami, 2022). This provides another mechanism for Ca_v_1.3 regulation (Jenkins et al., 2010; Tokumitsu and Sakagami, 2022).

Alternative splicing in the Ca_v_1.3 C-terminus affects its electrophysiological properties by reducing Ca^2+^-dependent inactivation of the Ca_v_1.3 channels (Tan et al., 2011). A recent study by Lu et al. showed that the C-terminus of Ca_v_1.3 undergoes cleavage and translocation to the nucleus, where it acts as a transcription factor that affects the function of Ca^2+^-activated K^+^ channels in atrial cardiomyocytes (Lu et al., 2015).

This review summarizes the functional role and regulation of Ca_v_1.3 in healthy and diseased hearts. Specifically, we provide current knowledge on Ca_v_1.3 regulation across different cardiac conditions and the resulting implications for diseases and potential novel therapies.

## Functional role of Ca_V_1.3 in the heart

In cardiac musculature, Ca_v_1.3 is involved in pacemaking and AV conduction of the heart (Mesirca et al., 2016b). Ca_v_1.3^−/−^ mice are deaf and exhibit bradycardia and arrhythmia resulting from sinoatrial (SA) node dysfunction (Platzer et al., 2000; Ortner, 2023). This is likely because of the crucial role that Ca_v_1.3 channels play in the diastolic depolarization of SA node pacemaker cells (Mangoni et al., 2003). In this regard, action potentials recorded from the SA nodes in Ca_v_1.3^−/−^ mice show a significant reduction in beating frequency and diastolic depolarization rate compared with Ca_v_1.3^+/−^ or wild-type littermates, suggesting that this decrease is intrinsic to the SA node (Zhang et al., 2002).

Another study reported that Ca_v_1.3 deficiency impaired intracellular Ca^2+^ ([Ca^2+^]_i_) dynamics by decreasing the frequency of local [Ca^2+^]_i_ release, eventually leading to dysfunctional synchronization (Torrente et al., 2016). Ca_v_1.3 appeared to stimulate and synchronize ryanodine receptor (RyR)-dependent [Ca^2+^]_i_ release during regular SA node pacemaker activity. Thus, Ca_v_1.3 plays dual roles by mediating inward I_CaL_ and stimulating RyR-dependent [Ca^2+^]_i_ release. This provides an additional pathophysiological mechanism for congenital SA node dysfunction and heart block linked to the loss of Ca_v_1.3 function in humans (Boutjdir, 2000; Torrente et al., 2016). Ca_v_1.3 was implicated as an essential molecular component of the voltage-dependent, dihydropyridine-sensitive Na^+^ current (I_st_), essential in SA node automaticity. Hence, I_st_ and I_CaL_ share Ca_v_1.3 as a common molecular determinant in the SA node, despite the relatively unknown molecular nature of I_st_ (Toyoda et al., 2017).

## Expression of Ca_V_1.3 in the heart

Ca_v_1.3 is generally less abundant than Ca_v_1.2, the predominant L-type Ca^2+^ channel in the heart and brain (Berger and Bartsch, 2014). The expression and localization of Ca_v_1.3 are developmentally regulated. Two forms of Ca_v_1.3 (250 kD and 190 kD) were observed, with the full-length (250 kD) channel protein predominant in the prenatal stages. Ca_v_1.3 channel protein was expressed in both atria and ventricles at fetal and neonatal stages but was absent in adult ventricles. The short form of Ca_v_1.3 is only expressed in the adult and is restricted to the atria (Qu et al., 2011).

The 190 kD form of Ca_v_1.3 represents the channel with a truncated C-terminus (Qu et al., 2011). Interestingly, this truncation of Ca_v_1.3 has been shown to translocate to the nucleus, functioning as a transcriptional regulator to alter the function of KCa2 in atrial myocytes. Nuclear translocation of the C-terminal domain of Ca_v_1.3 is modulated by [Ca^2+^]_i_. This results in a decrease in protein expression of myosin light chain 2, which interacts with and increases the membrane localization of KCa2 channels (Lu et al., 2015). Another study reported that the total and membrane expression of Ca_v_1.3 were significantly impaired by overexpression of the protein Snapin, resulting in the ubiquitin-proteasomal degradation of the channel and a consequent reduction of the total I_CaL_ densities (Sun et al., 2017).

In embryonic atrial cardiomyocytes, elevated Ca_v_1.3 expression was reported upon truncation and subsequent inhibition of Ca_v_1.2 in murine models. Western blot analysis indicated an increase of Ca_v_1.3 protein in the atrium, likely compensating for the functional loss of the truncated Ca_v_1.2 channel in these murine embryonic atrial cardiomyocytes by upregulating the Ca_v_1.3 channel (Ding et al., 2013).

**FIGURE 2:**
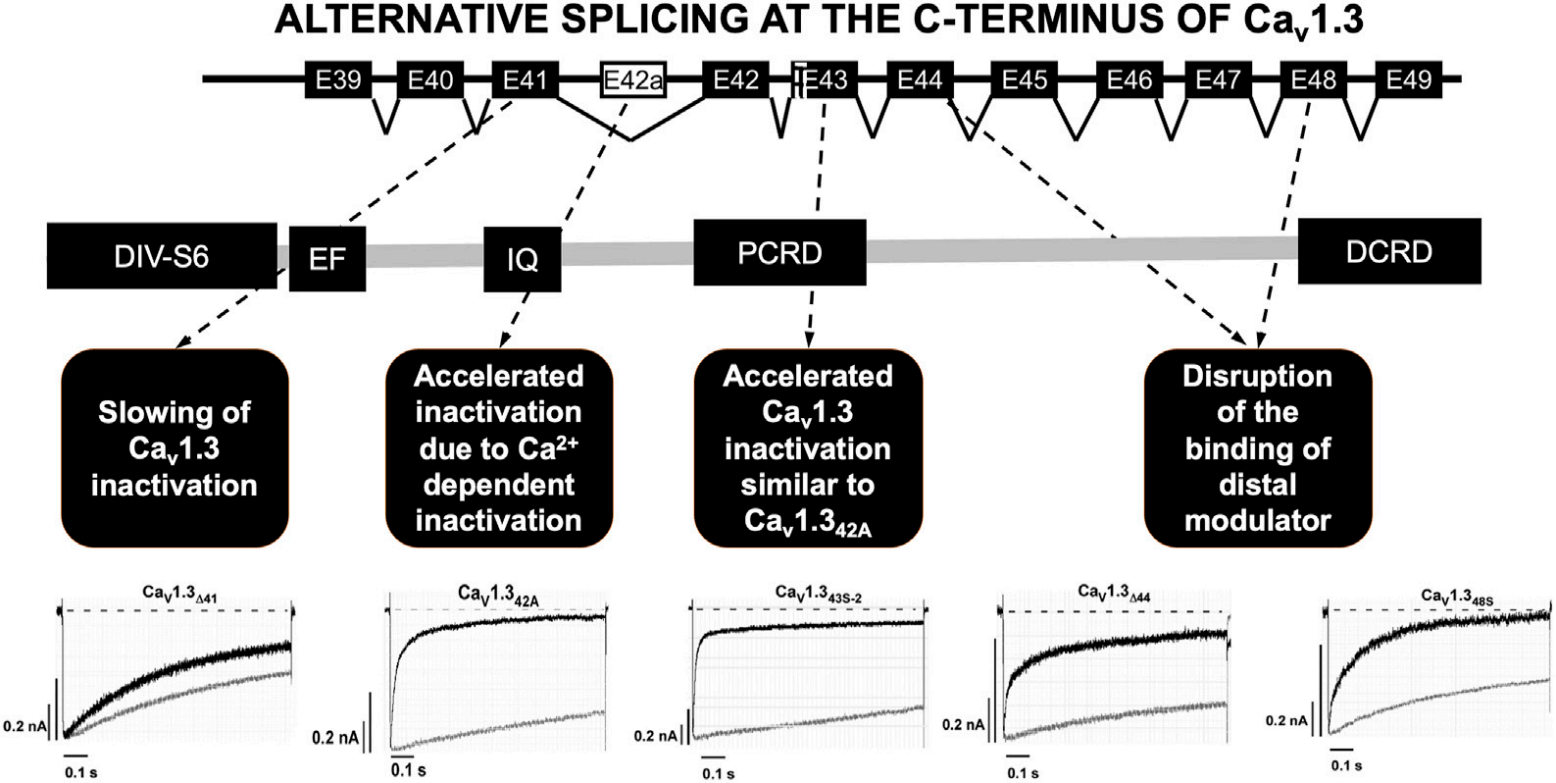
Alternative splicing at the C-terminus of Ca_v_1.3. Alternative splicing of exon 41 that removes the IQ motif resulted in a truncated Ca_v_1.3 protein with diminished inactivation. Splicing of exon 43 which causes a frameshift variant and is susceptible to accelerating the inactivation similar to Ca_v_1.3_42A_. Ca^2+^ current through Ca_v_1.3_42A_ channels increased at negative voltages, and inactivation was faster because of Ca^2+^-dependent inactivation. Splicing of exons 44 and 48 was an in-frame variant and caused disruption of the binding of distal modulator to the IQ domain. PCRD (proximal C-terminal regulatory domain), DCRD (distal C-terminal regulatory domain). Current tracing are from reference Tan et al., 2011.

The C-terminal part of the Ca_v_1.3 channel is encoded by exons 39 to 49 and it is the subject of intensive alternative splicing events that affect its function (Figure 2) (Singh et al., 2008; Scharinger et al., 2015; Hofer et al., 2021). Several splicing variants have been reported in the nervous system and their role in heart is not yet well elucidated (Hofer et al., 2021). The C-terminus is a strong target for alternative splicing due to the C-terminus gating modulator’s ability to prevent Ca^2+^ inactivation of the channels (Singh et al., 2008; Scharinger et al., 2015; Hofer et al., 2021). The long isoform (Ca_v_1.3_42L_) possesses all the regulatory domains, whereas two short splicing isoforms (Ca_v_1.3_42A_ and Ca_v_1.3_43S_) are characterized by the absence of the distal C-terminal regulatory domain or both proximal and distal C-terminal regulatory domains (Singh et al., 2008; Tan et al., 2011; Scharinger et al., 2015). Alternative splicing in the C-terminus of Ca_v_1.3 modulates its electrophysiological properties (Singh et al., 2008; Scharinger et al., 2015; Hofer et al., 2021). Activation of I_CaL_ through Ca_v_1.3_42A_ channels increased at negative voltages, and inactivation was faster due to enhanced Ca^2+^-dependent inactivation (Singh et al., 2008). Furthermore, the C-terminal modulator domain in the Ca_v_1.3_42_ isoforms competed with calmodulin (CaM) in regards to binding to the IQ domain (Kuzmenkina et al., 2019).

Alternative splicing was identified at four other different loci in the C-terminus of Ca_v_1.3. The splicing of exon 41 removes the IQ motif resulting in a truncated Ca_v_1.3 protein with diminished inactivation. Secondly, splicing of exon 43 results in a frameshift variant and is susceptible to increased inactivation similar to Ca_v_1.3_42A_. Lastly, the splicing of exons 44 and 48 in-frame causes disruption of the distal modulator binding to the IQ domain (Tan et al., 2011).

## Regulation of Ca_V_1.3 in the heart

### Regulation by PKA

**FIGURE 3:**
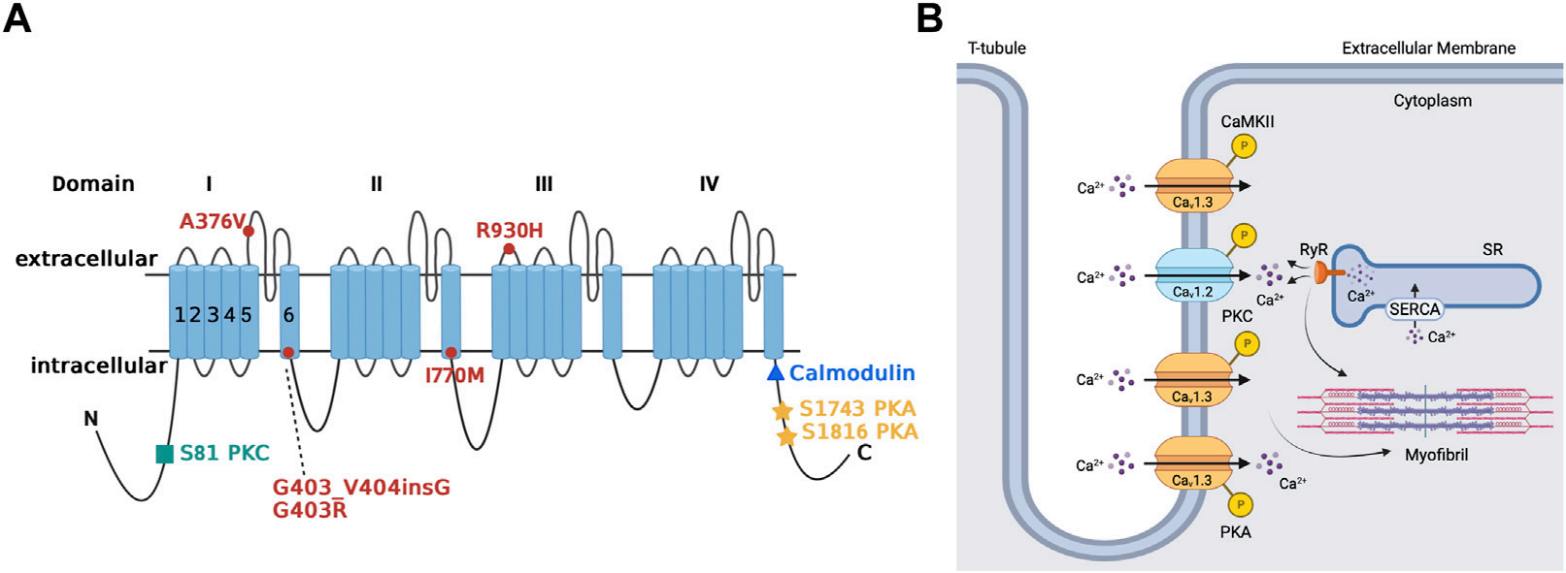
Regulation of Ca_v_1.3 L-type Ca^2+^ channel by protein kinase A, protein kinase C, and calmodulin-dependent protein kinase II. Panel **(A)** Schematic representation of the four homologous domains (I-IV) of the Ca_v_1.3 α_1_-subunit with 6 transmembrane segments (S1-S6) and N- and C- termini. Phosphorylation of the channel by PKA is at serine residues located at positions 1743 and 1816 of the C-terminus. PKC phosphorylates at the N-terminal domain at serine residue located at position 81. Calmodulin binding site is on the proximal C-terminus. Missense variant A376V and the founder variant G403_V404insG, as well as heterozygous non-synonymous variant R930H in *CACNA1D* gene have been associated with sinoatrial node dysfunction (Liaqat et al., 2019; Rinné et al., 2022). The missense variants G403R and I770M has been found in patients with atrioventricular node dysfunction (Scholl et al., 2013). Panel **(B)** The sketch summarizes the regulation of atrial Ca_v_1.3 channel by the different kinases, including PKA, PKC and CaMKII. Ca^2+^ entry through Ca_v_1.3 channel and subsequent Ca^2+^ release from RyR contributes to pacemaking, while Ca^2+^ entry through Ca_v_1.2 contributes to excitation-contraction coupling. PKA (protein kinase A), PKC (protein kinase C), SR (sarcoplasmic reticulum), SERCA (sarcoendoplasmic reticulum calcium ATPase), RyR (ryanodine receptor), P (phosphorylation site), CaMKII (calmodulin-dependent protein kinase II).

Ca_v_1.3 is upregulated through the PKA-cAMP pathway (Figure 3A) (Qu et al., 2005b; Ramadan et al., 2009). Specifically, Ramadan et al. showed 3 PKA consensus sites phosphorylated on the proximal C-terminus of the Ca_v_1.3 α_1_-subunit at serines 1743, 1816 and 1964 using mass spectrometry (Ramadan et al., 2009). Additional site-directed mutagenesis followed by patch clamp studies demonstrated that serines 1743 and 1816 were major functional PKA consensus sites while the phosphorylation of serine 1964 was not functionally relevant. The resulting PKA phosphorylation of Ca_v_1.3 increased channel activity in the SA node and atria (Qu et al., 2005b). The upregulation Ca_v_1.3 activity may account for as much as a 25% increase in total I_CaL_ (Qu et al., 2005b; Mahapatra et al., 2012; Vandael et al., 2013). On the other hand, decrease in PKA activity and subsequent downregulation of Ca_v_1.3 was reported in mice with a frameshift variant in the natriuretic peptide precursor A gene linked to AF (Menon et al., 2019). Collectively, these findings show that Ca_v_1.3 is a target for sympathetic control of heart rhythm *via* PKA.

### Regulation by PKC

There is limited available information about the regulation of Ca_v_1.3 by PKC in the heart. We showed that Ca_v_1.3 is inhibited through PKC activation by phosphorylation of its N-terminal domain (Figure 3A) (Baroudi et al., 2006). PKC activation reduces Ca_v_1.3 I_CaL_ by up to 50% by reducing the probability of Ca_v_1.3 to remain in an open state while increasing the likelihood and time spent in the closed state (Baroudi et al., 2006; Chahine et al., 2008). Interestingly, βIIPKC and εPKC are key PKC isozymes implicated in this regulatory mechanism, and serine 81 represents an essential site for PKC-mediated phosphorylation of Ca_v_1.3 (Baroudi et al., 2006). βIIV5-3 and εV1-2 peptides, which inhibit βIIPKC and εPKC, respectively, prevent the downregulation of Ca_v_1.3 by PKC. This further supports the importance of isozyme specific PKC in the regulation of Ca_v_1.3 and the essential consensus site at serine 81 in the downregulation of Ca_v_1.3 (Baroudi et al., 2006; Ferreira et al., 2012).

### Regulation by calmodulin

The prevailing understanding of CaM modulation of Ca_v_1.3 appears not to be limited to the binding of CaM to the C-terminus of the channel (Figure 3A) (Maier and Bers, 2002; Ben Johny et al., 2013). Johny et al. demonstrated that CaM might also bind to alternative sites on Ca_v_1.3, and subsequent binding of Ca^2+^ leads to alternative configurations of the CaM-Ca_v_1.3 complex, resulting in alternative forms of Ca^2+^-dependent inactivation (Ben Johny et al., 2013). Thus, in addition to the previously assumed association of CaM to the C-terminal IQ domain, the N-lobe may bind to the proximal Ca^2+^-inactivating region. Furthermore, if Ca^2+^ binds to the N-lobe of CaM, then it will bind onto the NSCaTE region of the Ca_v_1.3 channel (Dick et al., 2008; Ben Johny et al., 2013). Banerjee et al. reinforce this idea by demonstrating that CaM must bind to Ca_v_1.3 on both of its lobes to modulate Ca_v_1.3 to exit its preinhibited configuration (Banerjee et al., 2018). If CaM only binds on one of its sites to the Ca_v_1.3 channel, the channel will fail to be inhibited and operate as if CaM is not present. The C-terminal modulator domain in the distal C-terminus of the Ca_v_1.3 channel can interfere with CaM binding, resulting in subsequent inhibition of channel activity (Kuzmenkina et al., 2019). Ca_v_1.3 may also be modulated by CaMKII for increased channel activity (Maier and Bers, 2002; Gao et al., 2006; Tokumitsu and Sakagami, 2022). CaMKII-mediated phosphorylation of Ca_v_1.3 channel may result in the channels staying open for longer, and the increased channel activity manifests in an action potential that looks similar to an ascending staircase with multiple depolarizations from holding potential (Maier and Bers, 2002). The specific pathway of insulin growth factor 1 stimulates phospholipase C-ɣ to facilitate Ca^2+^ release from IP_3_-sensitive stores, thereby activating CaMKII and phosphorylating the Ca_v_1.3 channel (Gao et al., 2006).

## Ca_v_1.3 in heart disease

### Autoimmune-associated congenital heart block

Autoimmune-associated congenital heart block (aCHB) is an electrophysiological abnormality affecting the SA and AV nodes of structurally healthy hearts in fetuses and neonates. Clinical symptoms of aCHB include a spectrum of variations of sinus bradycardia and AV block. Of these variations, third-degree AV block is the most critical and lethal manifestation, having the greatest mortality rate (Lazzerini et al., 2017). aCHB is presumably acquired passively when the mother transmits anti-Ro/SSA auto-antibodies through the placenta to the fetus (Boutjdir, 2000; Lazzerini et al., 2017). Anti-Ro/SSA antibodies are the most common antibodies associated with aCHB (Karnabi et al., 2010; Qu et al., 2019). Qu et al. showed that human Ca_v_1.3 expression and associated electrophysiological activity were inhibited by anti-Ro/SSA positive IgG antibodies obtained from mothers that had children with aCHB (Qu et al., 2005a). This study also showed the ability of anti-Ro/SSA positive IgG to recognize the Ca_v_1.3 channel protein, which suggests cross-reactivity between the anti-Ro/SSA antibodies with the Ca_v_1.3 channel (Qu et al., 2005a; Qu and Boutjdir, 2012).

#### Sinoatrial node dysfunction: Autoimmune-associated sinus bradycardia

Autoimmune diseases provide an additional level of insight into the development of cardiovascular diseases since autoantibodies have been found to modulate cardiac electrophysiological activity (Qu et al., 2019). Voltage-gated L-type Ca^2+^ channels, specifically Ca_v_1.3, play a key role in the pathophysiology of cardiac arrhythmias in the presence of autoimmune antibodies such as anti-Ro/SSA and anti-La/SSB (Qu et al., 2019; Lazzerini et al., 2021). In particular, the anti-Ro/SSA antibodies interact with Ca_v_1.3 through a mechanism of direct channel inhibition (Qu et al., 2005a; Lazzerini et al., 2018; Qu et al., 2019). There are two types of anti-Ro/SSA antibodies: anti-52 kD and anti-60 kD, and they are formed as a result of an autoimmune response to the Ro/SSA antigen (Lazzerini et al., 2018; Qu et al., 2019). In experiments with immunized (Ro/SSA antigens) and non-immunized Ca_v_1.3^−/−^ mice, only immunized Ca_v_1.3^−/−^ mice displayed severe sinus bradycardia, significantly prolonged PR interval and significantly lower fetal parity when compared to non-immunized mice (Karnabi et al., 2011).

Additional experiments with pregnant mice that were injected with positive IgG from human mothers that had children with aCHB showed that the timing of immunization during gestation was important (Mazel et al., 1999). Although pups from the 8 days-, 11 days-, and 16 days-gestation groups showed electrocardiographic symptoms, pups that were at least 11 days along in gestation were more likely to develop a higher degree of sinus bradycardia and PR prolongation (Mazel et al., 1999; Boutjdir, 2000). Hu et al. showed that I_CaL_ was reduced in rabbit SA node cells following superfusion of maternal anti-Ro/SSA positive IgG from mothers who had children with aCHB resulting in slow spontaneous action potentials consistent with sinus bradycardia (Hu et al., 2004). As a potential therapy for aCHB, genetic or drug-induced deactivation of the muscarinic-gated K^+^ channel in Ca_v_1.3^−/−^ mice has been proposed to allow a net inward current to be maintained to prevent dysfunction of SA node pacemaking activity (Mesirca et al., 2016a). The role of autoimmune cardiac channelopathies involving Ca_v_1.3 in the development of cardiac arrhythmias represents an avenue for future investigation (Capecchi et al., 2019).

#### Atrioventricular node dysfunction: Autoimmune-associated atrioventricular block

A hallmark of aCHB is complete AV block almost always being accompanied by first-, second- or third-degree AV block (Boutjdir, 2000). In prospective studies of pregnancies in anti-Ro/SSA-positive women with no previously affected children, the risk of aCHB was estimated to be 2%–5%, whereas the risk of recurrence in mothers with a previously affected child increases to approximately 15%–20% (Boutjdir, 2000; Qu and Boutjdir, 2012). Treatment for aCHB includes dexamethasone, plasmapheresis, sympathomimetic and *in utero* cardiac pacing therapy, but none of these are definitive in successfully treating AV block (Boutjdir, 2000).

AV block was successfully induced in isolated Langendorff perfused human fetal hearts by purified IgG fractions and anti-52 kD Ro/SSA antibodies from mothers of children with aCHB (Boutjdir et al., 1997). Perfusion of maternal anti-Ro/SSA positive IgG into rat hearts resulted in the development of bradycardia associated with second-degree AV block, which then degenerated into complete AV block. Experiments have shown that anti-Ro/SSA antibodies led to a reduction of Ca_v_1.3 I_CaL_ by 35% in naive cardiomyocytes (Qu et al., 2019). Additionally, 14% of anti-Ro/SSA antibody-positive IgG was reactive with domain I of the extracellular S5-S6 loop in the pore-forming subunit of Ca_v_1.3 (Karnabi et al., 2010).

Altered electrophysiological activity was noted in mice and rabbits that were immunized with recombinant anti-Ro 52 antigen (Lazzerini et al., 2017; Lazzerini et al., 2019; Qu et al., 2019). Peak I_CaL_ recorded in human fetal cardiomyocytes was significantly inhibited by maternal 52 kD anti-Ro/SSA antibodies. Prior work has also shown that 52 kD anti-Ro/SSA showed more incidence of second-and third-degree AV block. In rabbit AV node cells, action potentials recorded following perfusion of human positive IgG showed significant reduction in the beating heart (Boutjdir, 2000). To culminate these findings, the inclusion of anti-Ro/SSA antibodies in isolated multicellular AV node preparations and Langendorff-perfused whole hearts resulted in bradycardia and AV block (Restivo et al., 2001; Qu and Boutjdir, 2012; Qu et al., 2019). IgG antibodies from mothers with children having aCHB reacted with the sarcolemma of human fetal cardiomyocytes and recognized Ca_v_1.3 subunits, as opposed to anti-Ro/SSA negative IgG antibodies from mothers that had healthy children (Qu et al., 2005a). Collectively, Ca_v_1.3 plays a critical role in the pathogenesis of conduction abnormalities seen in aCHB and can be a preferential target for novel therapies.

### Cardiac phenotypes in families with CACNA1D variants

#### Sinoatrial node dysfunction

Initially, no known human channelopathies were described for Ca_v_1.3 channels or its associated CACNA1D gene (Striessnig et al., 2010). Rinné et al. conducted a study on a three-generation Turkish family where whole genome sequencing was used to identify a variant of CACNA1D associated with SA dysfunction (Figure 3A) (Rinné et al., 2022) Specifically, examination of exon 22 on the CACNA1D gene led to characterization of the p (Arg930His) variant of the CACNA1D gene, which induces the alteration of the Ca_v_1.3 long isoform, thus resulting in loss of function of the channel which leads to SANDD. In this variant, there is a substitution of arginine for a histamine residue at position 930 of the extracellular linker between the S1 and S2 transmembrane segments of domain III on the Ca_v_1.3 channel, which is associated with the channel’s gating properties, resulting in loss of function (Rinné et al., 2022). Later, Baig et al. showed that an alteration in CACNA1D resulted in a glycine residue insertion near the Ca_v_1.3 pore, thus reducing Ca^2+^ entry, which became an identifying feature of SANDD (Baig et al., 2011). The CACNA1D gene holds significance in Pakistani lineages, where variants have been discovered that lead to inhibited Ca_v_1.3 function and possibly result in SA node dysfunction. Liaqat et al. identified and further characterized the founding variant p (G403_V404insG) and a new missense variant p (A376V), both of which exhibited a phenotype of SANDD (Liaqat et al., 2019).

#### Atrioventricular node dysfunction

The CACNA1D gene is also expressed in the AV node, meaning any variants have implication for Ca_v_1.3-related channelopathies in the AV node. In this regard, AV block was reported in members of a Turkish family that expressed the p (Arg930His) variant of the CACNA1D gene (Figure 3A) (Rinné et al., 2022). Scholl et al. were able to show that variants of the CACNA1D gene resulted in altered glycine (G403R) and isoleucine residues (I770M) in the S6 of Ca_v_1.3 domain I and II. This substitution increased channel activation and inhibited inactivation, leading to gain of function in Ca_v_1.3 for patients with aldosteronism; the cardiac implications are yet to be characterized and further clinical studies are warranted (Scholl et al., 2013).

### Atrial fibrillation

AF is the most common cardiac arrhythmia that contributes substantially to morbidity and mortality. The cellular mechanisms underlying AF are multifactorial. A reduction in I_CaL_ density was initially reported in atrial myocytes from patients with AF (Van Wagoner et al., 1999). Subsequently, atrial samples from patients with AF also showed a significant decrease in Ca_v_1.3 channel mRNA, pointing to a functional role for Ca_v_1.3 in AF development (Gaborit et al., 2005). Ca_v_1.3^−/−^ mice showed atrial electrical dysfunction and predisposition to the development of AF (Mancarella et al., 2008). The electrical abnormalities in the Ca_v_1.3^−/−^ mice were associated with reduced total I_CaL_ density, [Ca^2+^]_i_ transient, and dysfunctional [Ca^2+^]_i_ handling and atrial stimulation induced AF in Ca_v_1.3^−/−^ mice (Mancarella et al., 2008). Whole-cell I_CaL_ in atrial myocytes from Ca_v_1.3^−/−^ mice showed a significant depolarizing shift in voltage-dependent activation (Mancarella et al., 2008). In contrast, there were no significant differences in the I_CaL_ recorded from ventricular myocytes between wild-type and null Ca_v_1.3^−/−^ mice (Zhang et al., 2005). These data further support a potentially important role for Ca_v_1.3 in the development of AF.

Studies of molecular mechanisms for the role of Ca_v_1.3 in AF are still nascent. Reports show a reduction in ankyrin-B expression in the atria of patients with documented AF, suggesting that ankyrin-B was required for the membrane targeting and function of Ca_v_1.3 in atrial myocytes (Mesirca et al., 2021). Ankyrin-B was shown to associate with Cav1.3 directly. Loss of ankyrin-B in atrial myocytes resulted in decreased Ca_v_1.3 expression, membrane localization, and function, leading to shortened atrial action potentials and arrhythmias (Cunha et al., 2011; Wolf et al., 2013; Mesirca et al., 2021). In a subsequent study, reduced expression of Ca_v_1.3 paralleled with enhanced expression of Snapin was seen in atrial samples from AF patients (Sun et al., 2017). Upon further investigation, it appeared that Snapin downregulated Ca_v_1.3 membrane expression and promoted its degradation through the ubiquitin-proteasome pathway, thus functioning as a novel regulator for Ca_v_1.3 protein trafficking in atrial myocytes. Further mechanistic insights came from a study from our group, which showed that Ca_v_1.3 and the Ca^2+^ activated K^+^ channel SK4 were coupled in the atria and that Ca_v_1.3 deletion led to decreased SK4 mRNA and brain natriuretic peptide secretion from the atria (Srivastava et al., 2017). Regulation of atrial endocrine secretion by Ca_v_1.3 is a possible candidate pathway for generating cardiac arrhythmias such as AF. Particularly, cardiac Ca^2+^ (Ca_v_1.2/Ca_v_1.3) channel expression and I_CaL,_ along with the action potential durations, were significantly reduced in mice with frameshift human natriuretic peptide precursor A genes, providing further evidence of the significant role of Ca_v_1.3 in AF (Menon et al., 2019). Rose et al. showed that chronic iron overload reduced Ca_v_1.3 mRNA and I_CaL_, thereby suppressing channel function (Rose et al., 2011). They suggested this mechanism as a possible contributor to the development of AF.

### Heart failure

Heart failure (HF) is the heart’s inability to maintain adequate blood circulation to the body’s tissues or to pump out the venous blood returned to it by venous circulation (Dyck et al., 2022). There is substantial evidence that the contractility of failing human hearts is depressed (Houser and Margulies, 2003). As described above briefly, Ca^2+^ enters the cardiomyocytes *via* voltage-gated Ca^2+^ channels upon electrical excitation, which causes further Ca^2+^ release from the sarcoplasmic reticulum (Figure 3B). This raises free [Ca^2+^]_i_, thereby activating the contraction of cardiac tissue (Bers and Despa, 2006). Therefore, abnormalities in basal Ca^2+^ regulation and dysfunctional Ca^2+^ signaling cause contractile dysfunction and arrhythmias in pathophysiological conditions such as HF (Houser et al., 2000; Houser and Margulies, 2003).

However, the role of the L-type Ca^2+^ channels which provide Ca^2+^ entry to failing cardiomyocytes is unclear and controversial (Beuckelmann et al., 1992; Mukherjee et al., 1998; Benitah et al., 2010). Among L-type Ca^2+^ channels, Ca_v_1.2 is highly expressed in the ventricles; therefore, research on HF focused on Ca_v_1.2. No research except a recent study by Srivastava et al. has addressed the potential role of Ca_v_1.3 in HF (Srivastava et al., 2020). Published results on Ca_v_1.2 gene expression during HF are inconsistent and show either decreased or insignificant changes in mRNA levels (Takahashi et al., 1992; Schröder et al., 1998; Hong et al., 2012). Furthermore, there are contradictory reports where some studies observed an increase in I_CaL_ during HF, while others reported no changes (Schröder et al., 1998; Chen et al., 2002; Mørk et al., 2007).

**FIGURE 4:**
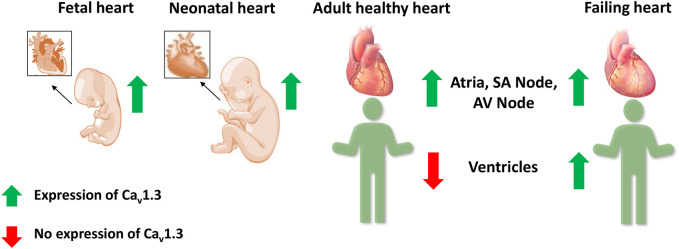
Expression of Ca_v_1.3 L-type Ca^2+^ channel in the fetal, neonatal, adult, and failing heart. Ca_v_1.3 is expressed in the supraventricular and ventricular tissue of the fetal and neonatal hearts. However in adult hearts, it is expressed only in the atria, sinoatrial (SA) node, and atrioventricular (AV) node, but not in the ventricles. Recent evidence suggests a Ca_v_1.3 *de novo* expression in the ventricles of adult failing hearts.

Several fetal genes, including the T-type Ca^2+^ channel Ca_v_3.1, are re-expressed during ventricular remodeling following experimental myocardial infarction in rats (Gidh-Jain et al., 1998; Huang et al., 2000). Atrial natriuretic peptide, hyperpolarization activated cyclic nucleotide gated potassium channel 4, β-myosin heavy chain, skeletal α-actin, and smooth muscle 22α are other genes that are also re-expressed in HF (Kuwahara et al., 2012; Nandi and Mishra, 2015; Sano et al., 2021). Therefore, Ca_v_1.3 was postulated as a possible candidate to be involved in ventricular remodeling in HF (Figure 4) (Nikolaidou et al., 2015; Menon et al., 2019; Zhang et al., 2020). In HF patients, we observed a 6.2-fold increase in Ca_v_1.3 mRNA levels and a 14.9-fold decrease in Ca_v_1.2 mRNA levels in failing hearts compared to healthy human left ventricular control tissue (Srivastava et al., 2020). This alteration was also reported for Ca_v_1.3 protein with western blots in seven failing hearts, demonstrating high expression of Ca_v_1.3 mRNA. A functional re-expression of Ca_v_1.3 might serve as a novel compensatory mechanism for the ventricle to improve cardiac function in HF (Ding et al., 2013; Srivastava et al., 2020; Torrente et al., 2020). Single-cell RNA sequencing data from human dilated and hypertrophic cardiomyopathy demonstrated that Ca_v_1.3 is highly expressed in activated fibroblasts (Chaffin et al., 2022). Thus, it cannot be excluded that the re-expression of Ca_v_1.3 in HF could also originate from cardiac fibroblasts, a common feature of ventricular cardiac remodeling in HF. To elucidate the potential role of Ca_v_1.3 in the ventricles of adult failing hearts, further investigations into its effect on I_CaL_ and inotropy in the ventricle will be required. It will be crucial to delineate the role of Ca_v_1.3 re-expression in HF for developing novel therapeutic interventions.

## Conclusion

The physiological role of L-type Ca^2+^ channels has been studied extensively, aided by generating gene knockout animal models. Given their crucial role in excitation-contraction coupling and maintaining a delicate balance of [Ca^2+^]_i_ in cardiomyocytes, there is a need for further investigation into these channels in diseased states, particularly Ca_v_1.3, as a therapeutic target. Table 1 reports published literature on the role of Ca_v_1.3 in SA node dysfunction, AV node conduction defects, AF, HF, and autoimmune cardiac channelopathies. However, most studies have not elucidated the molecular mechanisms that underlie disease progression and management. Downregulation or upregulation of Ca_v_1.3 observed in these various diseases will likely facilitate the maintenance of [Ca^2+^]_i_ and generating and regulating pacemaking. Hence, detailed mechanistic insights into the role of Ca_v_1.3 and its expression and function in the heart will assist in identifying new therapies targeted towards treating the aforementioned cardiovascular diseases.

**TABLE 1:** Summary of published literature on Ca_v_1.3 in SA/AV node dysfunction, atrial fibrillation, and heart failure.

Publication	Disease/Dysfunction	Summary
Platzer et al. (2000)	SA node dysfunction	Congenital deafness and SA node dysfunction in mice lacking Ca_v_1.3 L-type Ca^2+^ channels
Baig et al. (2011)	SA node dysfunction	Loss of Ca_v_ 1.3 (CACNA1D) function in a human channelopathy with bradycardia and congenital deafness
Liaqat et al. (2019)	SA node dysfunction	CACNA1D variants associated with SA node dysfunction and deafness in Pakistani families
Mangoni et al. (2003)	SA node dysfunction	Ca_v_1.3 channels contribute to diastolic depolarization in SA node pacemaker cells
Zhang et al. (2002)	SA node dysfunction	Role for Ca_v_1.3 in the generation of the spontaneous action potential in the SA node
Qu et al. (2005a)	SA node dysfunction	Ca_v_1.3^−/−^ mice develop sinus bradycardia and various degrees of atrio-ventricular block
Rose et al. (2011)	SA node dysfunction	Chronic iron overload reduces Ca_v_1.3 expression and associated electrical activity, potentially leading to sinus bradycardia
Karnabi et al. (2011)	SA/AV node dysfunction	Ca_v_1.3−/− mice infused with anti-Ro/SSA antibodies showed severe AV block and sinus bradycardia
Restivo et al. (2001)	SA/AV node dysfunction	Rabbit hearts infused with anti-Ro/SSA antibodies showed delayed action potentials in the sinoatrial junction, representing sinus bradycardia in addition to AV block
Mesirca et al. (2016a)	SA/AV node dysfunction	The muscarinic-gated K^+^ channel represents a good target for genetic inactivation or pharmacological inhibition to improve symptoms of in Ca_v_1.3−/− mice afflicted by sick sinus syndrome and AV block. Alternatives include selective suppression of Cav1.3-associated I_CaL_
Zhang et al. (2020)	AV node dysfunction	Ca_v_1.3^−/−^ mice show a significant decrease in the firing frequency of spontaneous action potentials suggesting an important role for Ca_v_1.3 in the automaticity of the AV node
Mancarella et al. (2008)	Atrial fibrillation	Ca_v_1.3^−/−^ mice are associated with reduced total I_CaL_ density, intracellular Ca^2+^ transient, and dysfunctional intracellular Ca^2+^ handling
Sun et al. (2017)	Atrial fibrillation	Reduced expression of Ca_v_1.3 paralleled with enhanced expression of Snapin was in atrial samples from AF patients
Gaborit et al. (2005)	Atrial fibrillation	Atrial samples from patients with AF show a significant reduction in Ca_v_1.3 channel mRNA
Zhang et al. (2005)	Atrial fibrillation	Total I_CaL_ in atrial myocytes from Ca_v_1.3^−/−^ mice shows a significant depolarizing shift in voltage-dependent activation
Cunha et al. (2011), Wolf et al. (2013)	Atrial fibrillation	Reduction in ankyrin-B expression in atria of patients with AF. Ankyrin-B is required for the membrane targeting and function of Ca_v_1.3 in atrial myocytes
Srivastava et al. (2017)	Atrial fibrillation	Elucidation of an atrial endocrine secretion pathway regulated by Ca_v_1.3 that is a possible candidate pathway involved in generation of cardiac arrhythmias such as AF
Menon et al. (2019)	Atrial fibrillation	Atrial natriuretic peptide (ANP) overexpressing mouse model is more prone to developing AF and shows a reduction in Ca_v_1.2/Ca_v_1.3 and I_CaL_
Schröder et al. (1998)	Heart failure	Increased availability and open probability of single L-type Ca^2+^ channels in failing human ventricles
Mørk et al. (2007)	Heart failure	Increased cardiomyocyte function and Ca^2+^ transients in mice during early congestive heart failure
Chen et al. (2002)	Heart failure	Density of L-type Ca^2+^ channels are reduced in failing ventricular cardiomyocytes but basal I_CaL_ density is maintained by increase in channel phosphorylation
Srivastava et al. (2020)	Heart failure	Ca_v_1.3 is expressed in HF patients and therefore is a possible candidate gene involved in ventricular remodeling in the failing heart

## Abbreviations

aCHB, autoimmune-associated congenital heart block; AF, atrial fibrillation; AV, atrioventricular; CaM, calmodulin; CaMKII, calmodulin-dependent protein kinase II; HF, heart failure; PKA, protein kinase A; PKC, protein kinase C; SA, sinoatrial; SANDD, sinoatrial node dysfunction and deafness.
